# MEOX1-mediated transcriptional regulation of circABHD3 exacerbates hepatic fibrosis through promoting m6A/YTHDF2-dependent YPEL3 mRNA decay to activate β-catenin signaling

**DOI:** 10.1371/journal.pgen.1011622

**Published:** 2025-03-18

**Authors:** Limin Chen, Hui Yang, Juan Wang, Haoye Zhang, Kangkang Fu, Yu Yan, Zhenguo Liu

**Affiliations:** 1 Department of Infectious Disease, The Third Xiangya Hospital, Central South University, Changsha, Hunan Province, P.R. China; 2 Hunan Key Laboratory of Viral Hepatitis, Xiangya Hospital, Central South University, Changsha, Hunan Province, P.R. China; Institut Jacques Monod, FRANCE

## Abstract

**Background:**

Hepatic fibrosis may progress to liver cirrhosis and eventually cause death. Epithelial-mesenchymal transition (EMT) of hepatocytes plays critical roles in hepatic fibrosis. Exploring the mechanisms underlying EMT is crucial for a better understanding of hepatic fibrosis pathogenesis.

**Methods:**

Hepatocyte EMT wad induced with TGF-β1 and evaluated by Western blotting and immunofluorescence staining. Methylated RNA immunoprecipitation (MeRIP) was applied to assess N6-methyladenosine (m6A) modification. RIP and RNA pull-down assays were performed to analyze the interaction between circABHD3, YTHDF2 and YPEL3 mRNA. MEOX1-mediated transcription of ABHD3 was examined by luciferase and chromatin immunoprecipitation (ChIP). Mice were intraperitoneally injected with CCl_4_ or treated with bile duct ligation (BDL) surgery for hepatic fibrosis induction. Liver injury and collagen deposition were examined with hematoxylin and eosin (HE), Masson, and Sirius Red staining. Alanine transaminase (ALT), aspartate transaminase (AST) and hydroxyproline (HYP) were examined using ELISA.

**Results:**

CircABHD3 was upregulated in *in vitro* and *in vivo* models of hepatic fibrosis and patients. Knockdown of circABHD3 inhibited TGF-β1-induced expression of fibrosis markers, EMT and mitochondrial impairment in hepatocytes. MEOX1 could directly bind to the promoter of ABHD3 to facilitate its transcription and subsequent circABHD3 generation. Knockdown of MEOX1 suppressed TGF-β1-induced EMT and mitochondrial impairment through suppression of circABHD3. CircABHD3 destabilized YPEL3 mRNA via promoting YTHDF2-dependent recognition of m6A-modified YPEL3 mRNA to trigger β-catenin signaling activation. Furthermore, circABHD3 silencing-mediated inhibition of EMT and mitochondrial impairment was counteracted by YPEL3 knockdown and activation of β-catenin signaling. Depletion of circABHD3 significantly reduced EMT, mitochondrial impairment and hepatic fibrosis via promoting YPEL3 expression and suppressing β-catenin signaling *in vivo*.

**Conclusion:**

MEOX1-mediated generation of circABHD3 promotes EMT and mitochondrial impairment by enhancing YTHDF2-mediated degradation of YPEL3 mRNA and activating downstream β-catenin signaling, thus exacerbating hepatic fibrosis.

## Author summary

Hepatic fibrosis is a pathological condition characterized by the excessive deposition of extracellular matrix (ECM) proteins within the liver that leads to liver dysfunction. Epithelial-mesenchymal transition (EMT) is proposed to contribute to fibrogenesis by enabling epithelial cells to acquire a fibroblastic phenotype and participate in the production of ECM components. Understanding the cellular and molecular mechanisms involved in hepatic fibrosis is crucial for the development of effective therapies to prevent or reverse this condition and its associated complications. Here, we describe a novel mechanism involving circular RNA and EMT in liver fibrosis. CircABHD3 promotes EMT and mitochondrial impairment via facilitating YTHDF2-mediated degradation of YPEL3 mRNA and activating downstream β-catenin signaling, thus exacerbating hepatic fibrosis. This finding is particularly important as it provides a novel insight into the pathogenesis of hepatic fibrosis and potential targets for anti-fibrosis treatment.

## Introduction

Hepatic fibrosis is characterized by excessive formation of extracellular matrix (ECM) proteins such as collagen and caused by many chronic liver conditions [1]. The major causes of hepatic fibrosis include fatty liver disorder, excessive drinking, autoimmune hepatitis and chronic hepatitis B or C virus infection. Hepatic fibrosis usually shows no or vague symptoms, which may cause delayed medical treatment for patients. Unfortunately, hepatic fibrosis can progress to a fatal liver cirrhosis, and liver transplantation may be the only option for many patients. Currently, the treatment for hepatic fibrosis primarily focuses on removing the cause of fibrosis such as anti-hepatitis virus and limiting alcohol consumption, anti-inflammation and liver protection [2]. Exploring the mechanisms underlying the pathogenesis of hepatic fibrosis is central for developing promising therapies.

Epithelial-mesenchymal transition (EMT), a process by which epithelial cells acquire properties of mesenchymal cells and lose intercellular adhesion and cell polarity [3], may be a key process in hepatic fibrosis. Various cells in the liver, such as bile duct cells and hepatocytes, can be differentiated into myofibroblasts via EMT [4]. Myofibroblasts are major ECM-generating cells, thereby contributing to the formation of liver fibrosis [5]. Transforming growth factor-β1 (TGF-β1) is a key regulator that activates the differentiation of liver stellate cells into myofibroblasts, resulting in fibrosis [6]. Inhibition of TGF-β1-mediated EMT in liver cells improves hepatic fibrosis [7,8]. Further investigations of the regulation of EMT are essential for a better understanding of the pathogenesis of hepatic fibrosis.

Wnt/β-catenin signaling is one of the most important pathways involved in the regulation of EMT [9,10]. Wnt/β-catenin also regulate the vimentin, collagen 1, and fibronectin in liver fibrosis [11]. In fibrosis, the Wnt signaling is active, the β-catenin/E-cadherin complex is disrupted, β-catenin translocates into the nucleus, and E-cadherin is degraded thus enhancing the loss of the epithelial phenotype [12]. Ge et al. reported that β-catenin was highly expressed in liver fibrosis [13]. Therefore, understanding the mechanisms underlying the regulation of β-catenin signaling in hepatic fibrosis is important. Glycogen synthase kinase 3β (GSK-3β), an evolutionarily conserved serine/threonine kinase, phosphorylates β-catenin at Ser33, Ser37 and Thr41 to promote its degradation, thus downregulating downstream EMT-related markers and inhibiting EMT [14,15]. Liu et al. observed that bone marrow mesenchymal stem cells (BMSCs) suppressed hepatic fibrosis through activation of GSK-3β and subsequent inhibition of β-catenin signaling [16]. Also, Yippee-like-3 (YPEL3) is emerging as a negative regulator of β-catenin signaling. Zhang et al. found that YPEL3 inhibited EMT and metastasis in nasopharyngeal carcinoma via suppressing β-catenin signaling [17]. YPEL3 inhibits β-catenin signaling to negatively regulate endometrial function [18]. The suppressive role of YPEL3 in β-catenin signaling indicates that YPEL3 may be involved in hepatic fibrosis. N6-methyladenosine (m6A) modification is an important posttranslational modification that controls RNA stability and fate [19]. We found through bioinformatics analysis that there was an m6A modification site on YPEL3 mRNA. However, m6A-mediated regulation of YPEL3 mRNA stability is unknown.

Circular RNAs, a novel class of non-coding RNAs with a closed loop, show important activity in hepatic fibrosis. Zhou et al. reported 69 differentially expressed circRNAs in hepatic fibrosis [20], suggesting that circRNAs may be regulators of hepatic fibrosis. For example, circFBXW4 and circPSD3 ameliorate liver fibrosis through various mechanisms [21,22]. Circular RNA α/β-hydrolase domain-containing 3 (circABHD3, hsa_circ_0047086) is significantly upregulated in hepatic fibrosis tissues, but its roles in EMT and hepatic fibrosis remain largely unknown. Besides, the upstream regulatory mechanism of circABHD3 generation also remains unclear. We identified two potential binding sites for Mesenchyme Homeobox 1 (MEOX1) in the promoter region of ABHD3, indicating the possibility that MEOX1 may be implicated in the regulation of ABHD3 transcription and circABHD3 generation.

In our study, we reported that MEOX1 bound to the promoter of ABHD3 to enhance circABHD3 generation. Subsequently, circABHD3 promoted YTHDF2-dependent recognition of YPEL3 mRNA to destabilize YPEL3 mRNA to reduce its expression and activate β-catenin signaling, thus exacerbating hepatic fibrosis. Our study sheds novel light on the pathogenesis of hepatic fibrosis and provides potential therapeutic targets such as circABHD3, YPEL3 and β-catenin signaling.

## Methods

### Ethics approval and consent to participate

Written informed consent was provided by patients and volunteers, and our study was approved by the Ethics Committee of the Third Xiangya Hospital, Central South University. Animal procedures were approved by the Animal Care and Use Committee of the Third Xiangya Hospital, Central South University (the approval number: 2021-S278).

### Clinical specimens

Liver biopsy samples were collected from eight patients with hepatic fibrosis at the Third Xiangya Hospital, Central South University and stored at -80°C for RNA extraction. All patients included in this study were ≥18-year-old and did not receive any treatment. Patients with cancers, organ transplantation, pregnancy, drug and alcohol abuse and severe other diseases are excluded. We also collected liver samples from eight transplant donors as controls. Written informed consent was provided by patients and volunteers, and our study was approved by the Ethics Committee of the Third Xiangya Hospital, Central South University (the approval number: 2021-S278).

### Cell culture and treatment

Human and murine liver cell lines THLE-2 and AML12 were obtained from the American Type Culture Collection (Manassas, VA, USA) and maintained in Dulbecco’s Modified Eagle Medium (DMEM, Gibco, Carlsbad, CA, USA) supplemented with 10% fetal bovine serum (FBS, Gibco). To establish a cell model of hepatic fibrosis, cells were treated with TGF-β1 (PeproTech, Cranbury, NJ, USA) at 5 ng/mL for 48 h [23]. For inducing the activation of β-catenin signaling, cells were treated with lithium chloride (LiCl) at 50 mM for 16 h [24].

### Cell transfection

The sequences of circABHD3 and MEOX1 were inserted into the pLCDH-ciR vector (Geneseed Biotech, Guangzhou, China) and pcDNA3.1 Mammalian Expression Vector (Thermo Fisher Scientific, Waltham, MA, USA) for overexpression, respectively. YTHDF2 (shYTHDF2), circABHD3 (shcircABHD3), YPEL3 (shYPEL3), MEOX1 (shMEOX1#1, #2 and #3) and scramble shRNA (shNC) lentiviral constructs were provided by GenePharma (Shanghai, China). THLE-2 and AML12 cells were transiently transfected with shNC, shcircABHD3, shYTHDF2, shYPEL3, shMEOX1, circABHD3, MEOX1 or empty vector with Lipofectamine 2000 (Thermo Fisher Scientific). After 48 h, cells were detached and washed for subsequent experiments.

### Nuclear and cytoplasmic RNA extraction

Nuclear and cytoplasmic RNA of THLE-2 cells were extracted separately using Cytoplasmic and Nuclear RNA Purification Kit (Norgen, Thorold, ON, Canada) and reversely transcribed into cDNA. The abundance of glyceraldehyde-3-phosphate dehydrogenase (GAPDH), U6 snRNA, MATAL1, Mt-COI and circABHD3 was examined by qRT-PCR.

### CircABHD3 characterization

Total RNA was extracted from THLE-2 cells and treated with RNase R (5 U/µg, Abcam, Cambridge, UK) at 37°C for 1h. The remaining of circABHD3 and ABHD3 mRNA was analyzed by qRT-PCR. THLE-2 cells were treated with actinomycin D (a transcription inhibitor, Selleck, Shanghai, China) at 4 μg/mL for 0, 4, 8,16 and 24 h, and RNA was isolated and subjected for quantitative reverse transcription PCR (qRT-PCR) analysis of circABHD3 and ABHD3 mRNA. Divergent primers were designed for amplifying circABHD3, and the junction site of circABHD3 was identified by Sanger sequencing (Sangon, Shanghai, China).

### Methylated RNA immunoprecipitation (MeRIP)

The m6A level of YPEL3 mRNA was analyzed using the Magna MeRIP m6A Kit (Millipore, Billerica, MA, USA). Briefly, RNA was extracted from THLE-2 and AML12 cells and quantified with the Nanodrop spectrophotometer. Magnetic beads/anti-m6A antibody complex was prepared by adding 10 µg of the anti-m6A antibody into magnetic beads and incubating for half an hour. During preparing the magnetic beads/antibody complex, RNA (200 µg) was fragmented to approximately 100 nt. MeRIP Reaction Mixture was prepared following the manual, and the magnetic beads/antibody complex was mixed with MeRIP Reaction Mixture thoroughly for 2 h of incubation. Subsequently, the sample was placed on the magnet and washed twice. The immunoprecipitated m6A-modified RNAs were recovered and subjected for qRT-PCR analysis of YPEL3 mRNA.

### YPEL3 mRNA stability analysis

To analyze the stability of YPEL3 mRNA, after treatment, THLE-2 and AML12 cells were treated with actinomycin D at 4 μg/mL for 0, 4, 8 or 12 h and resuspended in Trizol reagent for RNA isolation. Subsequently, RNA was reversely transcribed, and the remaining of YPEL3 mRNA was detected by qRT-PCR and normalized to its remaining at 0 h.

### Fluorescence in situ hybridization (FISH) and immunofluorescence (IF) staining

Coverslip-bound cells were fixed in 3.7% paraformaldehyde solution for 10 min and immersed in 0.5% Triton X-100 for 20 min. For IF staining, cells were blocked in 5% bovine serum albumin (BSA) solution and incubated with primary antibodies against E-cadherin (1:1000, mouse, ab231303, Abcam) and N-cadherin (1: 500, rabbit, ab18203, Abcam) overnight. Cells were rinsed and incubated with Alexa Fluor 488- (1:2000, goat anti-mouse, Thermo Fisher Scientific) and Alexa Fluor 647-conjugated (1:2000, goat anti-rabbit, Thermo Fisher Scientific) secondary antibodies for 1 h. For combined FISH and IF staining, cells were treated with proteinase K (Thermo Fisher Scientific) at 8 µg/µL for 30 min and pre-hybridized for 2 h. Subsequently, cells were hybridized with Cy3-labelled circABHD3 probes (40 nM) at 56°C overnight. After rinse, cells were blocked and incubated with a YTHDF2 antibody (1:500, rabbit, Abcam) for 1 h in the dark. Cells were washed and incubated with Alexa Fluor 594-conjugated secondary antibody (1:2000, goat anti-rabbit, Thermo Fisher Scientific) for 1 h. Finally, cells were stained with 4’,6-diamidino-2-phenylindole (DAPI, Solarbio, Beijing, China) and mounted for examination under a fluorescence microscope.

### RNA immunoprecipitation (RIP)

Imprint RNAImmunoprecipitation (RIP) Kit (Sigma-Aldrich, Saint Louis, MO, USA) was used for RIP assays according to the manual. In brief, cells were scraped, resuspended in lysis buffer supplemented with protease and ribonuclease inhibitors and incubated for 20 min on ice followed by supernatant collection. For RIP, a YTHDF2 antibody (10 µg, rabbit, ab220163, Abcam) was pre-bound to protein A magnetic beads following the manual. Antibody-coated magnetic beads were mixed with the supernatants in IP buffer and incubated with rotation at 4°C overnight for immunoprecipitation of protein-RNA complexes. Magnetic beads were washed, and RNA was purified and subjected to qRT-PCR analysis of circABHD3 and electrophoresis.

### Dual-luciferase reporter assay

Site 1 (-909 to -915 bp) and Site 2 (-1018 to -1024 bp) in ABHD3 promoter, ABHD3 promoter containing wildtype Site 2 (pro-WT) or mutant Site 2 (pro-MUT), wildtype YPEL3 and A539-mutated YPEL3 were cloned into the pGL3 vector (Promega, Madison, WI, USA) as luciferase reporters. THLE-2 and AML12 cells were transfected with indicated luciferase reporters and the MEOX1-overexpressing pcDNA3.1 vector or circABHD3-overexpressing vector. Empty vector was used as a negative control. After 48 h, the luciferase activity was examined with Dual-Glo Luciferase System (Promega).

### Chromatin Immunoprecipitation (ChIP)

THLE-2 and AML12 cells were crosslinked in 1% formaldehyde solution, washed, and lysed in lysis buffer on ice. Cell lysates were collected and sonicated to obtain DNA fragments with a length of ~500 bp. Anti-MEOX1 antibody (5 µg, sc-398845, Santa Cruz Biotechnology, Dallas, TX, USA) and isotype control IgG were added into DNA fragments, and samples were incubated for 16 h at 4°C. Protein A/G Magnetic Beads (Abcam) were added into samples, and samples were incubated for 2 h. DNA was recovered and subjected to quantitative PCR.

### RNA Pull-down assays

For RNA pull-down, biotin-conjugated circABHD3 and scramble sequences (Genepharma) were mixed with the supernatants and incubated at 4°C for 5 h. The mixture was then mixed with streptavidin-conjugated magnetic beads and incubated for 3 h. Magnetic beads were washed, and protein-RNA complexes pulled down by the circABHD3 probe were used for analyzing the abundance of YTHDF2 (1:1000, ab220163, Abcam).

### A mouse model of hepatic fibrosis

Wildtype and circABHD3 knockout C57BL/6 mice (8-week-old, male, Cyagen Biosciences Inc., Santa Clara, CA, USA) were divided into four groups: Vehicle, CCl_4_, CCl_4_ + circABHD3-KO, CCl_4_ + circABHD3-KO + shNC and CCl_4_ + circABHD3-KO + shYPEL3. For CCl_4_ treatment, mice were intraperitoneally injected with CCl_4_ (5%, 200 µL/kg body weight, Sigma-Aldrich) in olive oil twice a week for six weeks [25]. Olive oil was injected into mice in the Vehicle group. shYPEL3 lentiviral construct (Genepharma) was transduced into HEK293T cells for packaging lentiviral particles. Lentivirus was harvested after 48 h, filtered, and intravenously injected into mice (5 × 10^8^ pfu/mouse) prior. Finally, serum was collected, and mice were sacrificed. The livers were excised for Western blotting and histology and immunohistochemistry staining. Animal procedures were approved by the Animal Care and Use Committee of the Third Xiangya Hospital, Central South University (the approval number: 2021-S278).

### Bile duct ligation (BDL) surgery

BDL surgery was performed as previously described with minor modification [26,27]. Briefly, mice were anesthetized via isoflurane inhalation, and the common bile duct was isolated from the flanking portal vein after a midline abdominal incision was made. Then, a non-resorbable suture (silk 5-0) was used to occlude the bile duct via a double ligature. Mice received intraperitoneal injection of tramadol at 0.25 mg/kg. Mice were sacrificed after 4 weeks.

### Histology and immunohistochemistry (IHC) staining

Liver samples from CCl_4_-induced hepatic fibrosis mice were immersed in 4% paraformaldehyde solution and fixed overnight at 4°C. Livers were dehydrated, embedded in paraffin, and cut into 5-µm slices. Slices were then dewaxed and rehydrated. Subsequently, standard hematoxylin and eosin (H&E), Masson’s trichrome and Sirius Red staining were applied to examine liver injury, collagen deposition and fibrotic area in the livers. For IHC staining, antigen was retrieved, and slices were sequentially blocked in 0.5% hydrogen peroxide solution and 5% BSA solution and incubated with rabbit α-smooth muscle actin (α-SMA, 1:100, ab5694), β-catenin (1:200, ab223075), E-cadherin (1:100, 3195, Cell Signaling Technology, Danvers, MA, USA) or N-cadherin (1:200, ab18203) antibodies overnight. Next day, a horseradish peroxidase (HRP)-conjugated goat anti-rabbit secondary antibody (1:5000, ab205718) was used to incubate sections. Diaminobenzidine (DAB, Solarbio) was applied for visualizing signals prior to hematoxylin staining. Slices were mounted for imaging. Antibodies use in IHC assays were purchase from Abcam unless otherwise indicated.

### JC-1 staining

Mitochondrial membrane potential was evaluated by JC-1 (Thermo Fisher Scientific) staining. JC-1 is a cationic dye that exhibits potential-dependent accumulation in mitochondria, indicated by a fluorescence emission shift from green (~525 nm, JC-1 monomer) to red (~590 nm, JC-1 aggregate). Thus, mitochondrial depolarization can be indicated by a decrease in the red/green fluorescence ratio. Briefly, cells were transfected and treated as indicated. After wash, cells were stained with JC-1 at 2 μM for 30 min and washed three times in PBS for imaging.

### Enzyme-Linked Immunosorbent Assay (ELISA)

The levels of aspartate aminotransferase (AST) and alanine aminotransferase (ALT) in the serum were determined using AST and ALT assay kits provided by Nanjing Jiancheng Bioengineering Institute (Nanjing, Jiangsu, China) following the manufacturer’s recommendations. The concentration of hydroxyproline (HYP) in the livers was measured using the HYP assay kit (Nanjing Jiancheng Bioengineering Institute). The levels of 8-hydroxy 2 deoxyguanosine (8-OHdG) in cell extracts and were examined with 8-hydroxy 2 deoxyguanosine ELISA Kit (ab201734, Abcam).

### Reactive oxygen species (ROS)

Cells were transfected and treated as indicated, washed, seeded in 96-well plates, and stained with DCFH-DA Redox Probe (G-Biosciences, St. Louis, MO, USA) at 10 μM for 30 min. Then, cells were washed three times in PBS, and the fluorescence was measured (Ex: 495 nm; Em: 529 nm) and normalized to the Control sample.

### Transmission electron microscopy (TEM)

TEM was performed as previously described [28]. Briefly, liver samples were collected from mice and immersed in 2.5% glutaraldehyde. Subsequenlty, samples were washed in PBS, fixed in 1% osmium tetraoxide for 1 h and dehydrated in gradient ethanol solutions. Then, samples were embedded, and ultrathin sections were prepared and stained with uranyl acetate and lead citrate for imaging under a transmission electron microscope.

### RNA extraction and qRT-PCR

Liver samples were homogenized, and Trizol reagent (Beyotime, Shanghai, China) was added into the homogenates for RNA extraction. THLE-2 and AML12 cells were resuspended in Trizol reagent, and total RNA was extracted followed by RNA quantification with the Nanodrop spectrophotometer (Thermo Fisher Scientific). RNA was then reversely transcribed into cDNA with HiScript II 1st Strand cDNA Synthesis Kit (Vazyme, Nanjing, Jiangsu, China), which was subjected to quantitative PCR using SYBR Green Master Mix (Vazyme) for analyzing the abundance of circABHD3, ABHD3, MEOX1, YPEL3, YTHDF2, Drp1, Fis1, OPA1 and Mfn1. Gene expression was normalized to GAPDH and analyzed using the 2^−∆∆Ct^ method.

### Western blotting

Liver tissues were homogenized, resuspended in lysis buffer, and incubated at 4°C for 2 h prior to supernatant collection. THLE-2 and AML12 cells were lysed in lysis buffer on ice, and supernatants were collected after centrifugation. BCA Protein Assay Kit (Solarbio) was used to quantify protein concentration. Protein (50 µg) was electrophoresed and transferred to polyvinylidene fluoride (PVDF) membranes, and membranes were blocked and incubated with rabbit antibodies against α-SMA (1 µg/mL, ab5694, Abcam), Collagen type I alpha 1 chain (COL1A1, 1:1000, ab260043, Abcam), Snail (1:1000, 3879, Cell Signaling Technology, Danvers, MA, USA), E-cadherin (1:1000, 3195, Cell Signaling Technology), N-cadherin (1 µg/mL, ab18203, Abcam), Vimentin (1:1000, ab92547, Abcam), YTHDF2 (1:1000, ab220163, Abcam), GSK-3β (1:500, ab131356, Abcam), β-catenin (1 µg/mL, ab223075, Abcam), phospho-β-catenin (1:1000, 9561, Cell Signaling Technology), MEOX1 (1:500, PA5-21037, Thermo Fisher Scientific), YPEL3 (1:1000, PA5-34349, Thermo Fisher Scientific) and GAPDH (1:2500, ab9485, Abcam) overnight. Membranes were washed and incubated with an HRP-conjugated goat anti-rabbit secondary antibody (1:25000, ab205718, Abcam) for 1 h. Bands were visualized with enhanced chemiluminescence (ECL) substrate and analyzed using Image J software.

### Statistical analysis

Results from at least three independent assays were expressed as mean ± standard deviation (SD). The variance of two and more groups was analyzed with the Student’s t test and One-way analysis of variance (ANOVA). *P* < 0.05 was statistically significant.

## Results

### CircABHD3 was upregulated in hepatic fibrosis patients and research models

**Fig 1:**
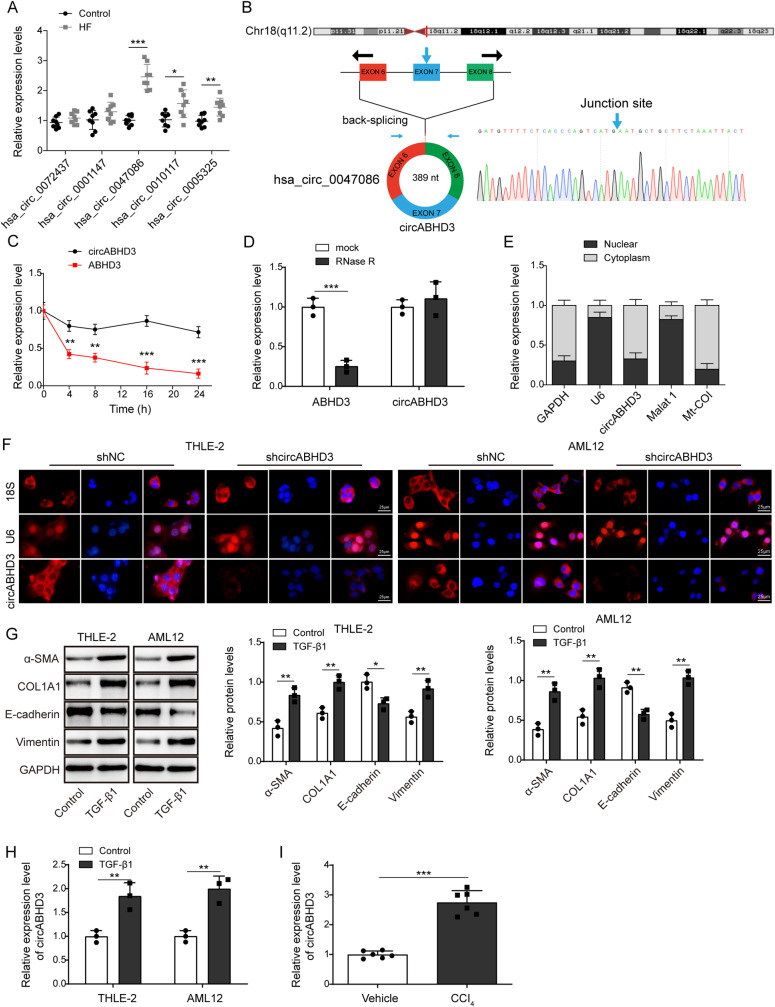
CircABHD3 was upregulated in hepatic fibrosis. (A) qRT-PCR analysis of hsa_circ_0072437, hsa_circ_0001147, hsa_circ_0047086, hsa_circ_0010117 and hsa_circ_0005325 in liver samples from hepatic fibrosis (n = 8) and control (n = 8) patients. (B) Genomic loci of circABHD3 and its formation via back-splicing. The junction site was identified by Sanger sequencing. (C and D) The abundance of circABHD3 and ABHD3 mRNA after actinomycin D and RNase R treatment (n = 3). (E) The abundance of circABHD3, MALAT1 (the nuclear reference), U6 (the nuclear reference), Mt-COI (the cytoplastic reference), and GAPDH (the cytoplastic reference) in the nucleus and cytoplasm. (F) THLE-2 and AML12 cells were transfected with shNC or shcircABHD3, and the localization of U6 snRNA (red, the nuclear reference), 18s rRNA (red, the cytoplastic reference) and circABHD3 (red) was examined by FISH. DAPI was used to stain the nuclei (blue). Scale bar, 25 µm. (G) THLE-2 and AML12 cells were treated with TGF-β1 at 5 ng/mL for 48 h. Protein levels of α-SMA, COL1A1, E-cadherin, and Vimentin were detected with Western blotting (n = 3). (H and I) qRT-PCR analysis of circABHD3 in TGF-β1 or vehicle-treated cells (n = 3) and CCl_4_ or vehicle-treated mice (n = 6). **P < 0.01 and ***P < 0.001.

As a previous study reported the upregulation of hsa_circ_0072437 (PARP8), hsa_circ_0001147 (RBM39), hsa_circ_0047086 (ABHD3), hsa_circ_0010117 (SPEN) and hsa_circ_0005325 (UBA2) in hepatic fibrosis tissues [29], we examined their expression in liver tissues from control donors and hepatic fibrosis patients, and found that hsa_circ_0047086 (circAHBD3) showed highest expression (Fig 1A). Therefore, we focused circABHD3 in this study. As shown in Fig 1B, circABHD3 (hsa_circ_0047086) is 389 nucleotides in length and originates from the exon 6 to 8 of *ABHD3* gene, and the back-splicing site was identified through Sanger sequencing. Actinomycin D, a transcription inhibitor, and RNase R were used to examine the stability of circABHD3. Compared to ABHD3 mRNA, circABHD3 showed high stability after actinomycin D treatment and excellent resistance to RNase R digestion (Fig 1C **and** 1D), exhibiting a typical stable characteristic of circRNA. Moreover, we found that circABHD3 mainly localized in the cytoplasm by nucleus and cytosol separation and FISH assays in THLE-2 and AML12 cells (Fig 1E **and** 1F). Compared to the signals of U6 snRNA and 18s rRNA, the signal of circABHD3 was largely diminished after circABHD3 knockdown (Fig 1F). We further found that fibrotic markers α-SMA and COL1A1 and the EMT-related marker Vimentin were upregulated but E-cadherin was downregulated by TGF-β1 induction (Fig 1G). Subsequently, we examined the expression levels of circABHD3 both in *vivo* and in *vitro* models of liver fibrosis. We found that TGF-β1 could induce significant expression of circABHD3 in hepatocyte cells, and in the liver tissue of mice with CCl_4_-induced fibrosis, circABHD3 expression was also significantly increased (Fig 1H **and** 1I). Moreover, neither TGF-β1 nor CCl_4_ treatment affect the levels of ABHD3 mRNA, the precursor of circABHD3 (S1A **and** S1B Fig). These data indicated that circABHD3 might be involved in the pathogenesis of fibrosis.

### Knockdown of circABHD3 significantly suppressed TGF-β1-induced EMT and mitochondrial impairment in hepatocytes

**Fig 2:**
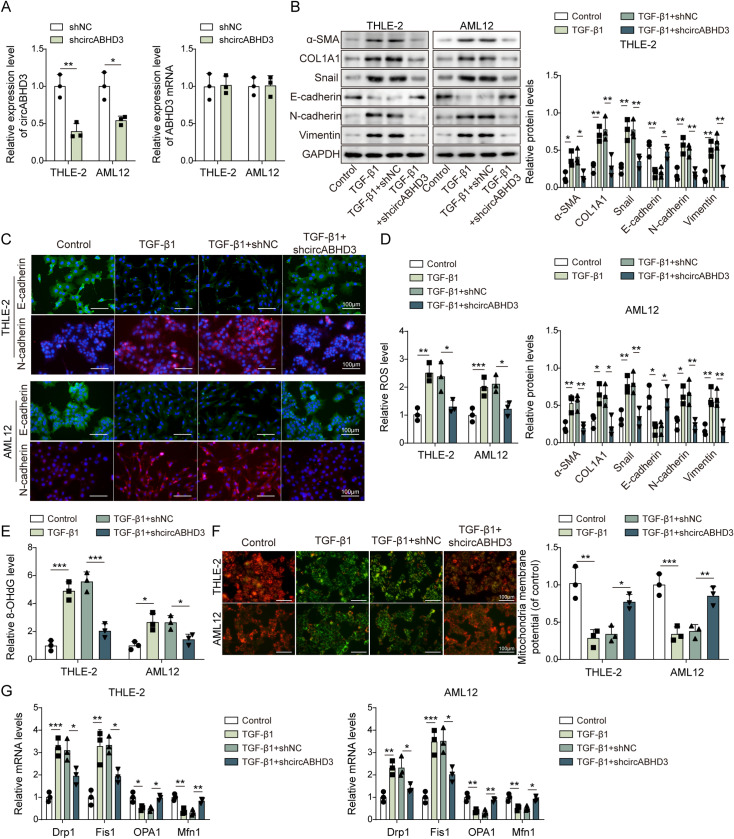
Knockdown of circABHD3 significantly suppressed TGF- **β1-induced EMT and mitochondrial impairment in hepatocytes.** THLE-2 and AML12 cells were treated with TGF-β1 at 5 ng/mL for 48 h and divided into four groups: Control, TGF-β1, TGF-β1 + shNC and TGF-β1 + shcircABHD3. (A) The levels of circABHD3 and ABHD3 mRNA in THLE-2 and AML12 cells were detected by qRT-PCR (n = 3). (B) Protein levels of α-SMA, COL1A1, Snail, E-cadherin, N-cadherin, and Vimentin were detected with Western blotting (n = 3). GAPDH was a loading control for normalization. (C) IF staining of E-cadherin (green) and N-cadherin (red). DAPI was used to stain the nuclei (blue). Scale bar, 100 µm. (D) The ROS level was determined (n = 3). (E) The level of 8-OHdG was examined (n = 3). (F) Mitochondrial membrane potential was examined with JC-1 staining (n = 3). JC-1 monomer, green; JC-1 aggregate, red. Scale bar, 100 µm. (G) The expression of Drp1, Fis1, OPA1 and Mfn1 was analyzed by qRT-PCR (n = 3). *P < 0.05 and **P < 0.01.

Knockdown of circABHD3 was performed in THLE-2 and AML12 cells (Fig 2A), which was followed by treatment with TGF-β1 to induce hepatic fibrosis to assess the impact of circABHD3 on the progression of liver fibrosis in these cell models. CircABHD3 was efficiently knocked down, and no reduction in the level of ABHD3 was observed in THLE-2 and AML12 cells (Fig 2A). Results showed that the expression of EMT-related markers, such as Snail, N-cadherin, and Vimentin, as well as fibrotic markers α-SMA and COL1A1, was upregulated by TGF-β1 induction, while E-cadherin was downregulated (Fig 2B). However, knockdown of circABHD3 abolished these effects (Fig 2B). IF staining showed that N-cadherin was upregulated but E-cadherin was downregulated, which was reversed by circABHD3 knockdown (Fig 2C). Moreover, TGF-β1-induced increased levels of ROS and 8-OHdG, a DNA damage marker, were inhibited by circABHD3 knockdown (Fig 2D **and** 2E). In addition, hepatic fibrosis is associated with mitochondrial dysfunction, which can lead to hepatocyte damage, immune cell activation, inflammation, and trans-differentiation of hepatic stellate cells [30–32]. We found that TGF-β1-induced increased JC-1 monomer and decreased JC-1 aggregate in TGF-β1-treated THLE-2 and AML12 cells were reduced by circABHD3 knockdown, suggesting that circABHD3 knockdown suppressed TGF-β1-induced impaired mitochondrial membrane potential (Fig 2F). Subsequently, we examined the expression of the mitochondrial fission regulators Drp1 and Fis1 and fusion regulators OPA1 and Mfn1. TGF-β1-induced upregulation of Drp1 and Fis1 and downregulation of OPA1 and Mfn1 were reversed by knockdown of circABHD3 (Fig 2G). In aggregate, our data indicated that the inhibition of circABHD3 suppressed TGF-β1-induced EMT and mitochondrial dynamic imbalance in hepatocyte cells.

### MEOX1 facilitated circABHD3 expression via binding to the promoter of ABHD3 in THLE-2 and AML12 cells

**Fig 3:**
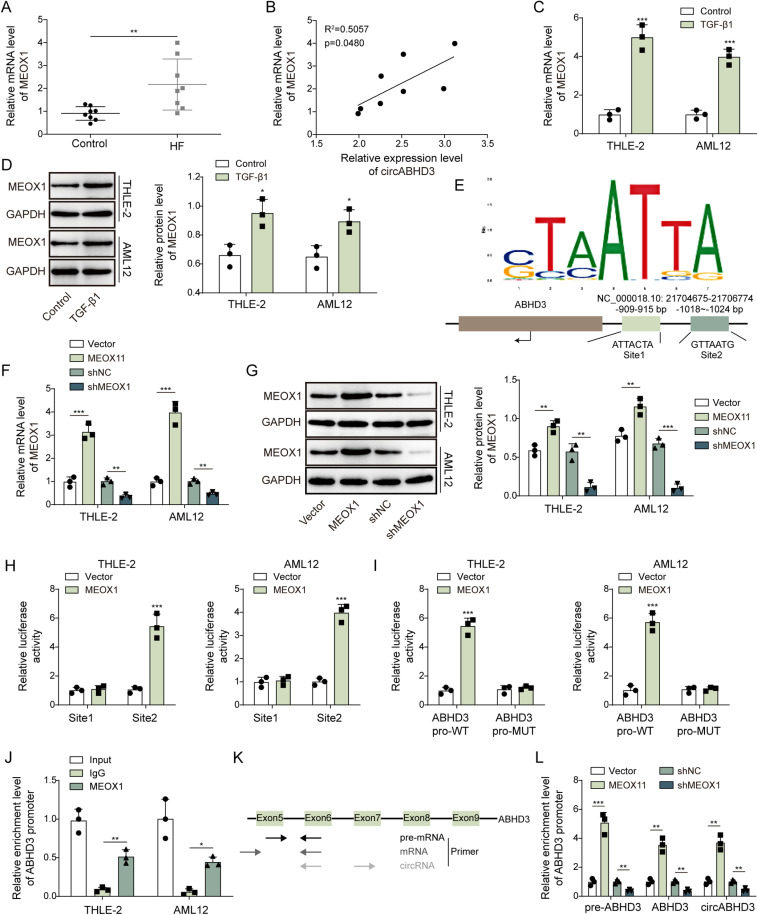
MEOX1 facilitated circABHD3 expression via binding to the promoter of ABHD3 in THLE-2 and AML12 cells. (A) qRT-PCR analysis of MEOX1 mRNA in liver samples from hepatic fibrosis (n = 8) and control (n = 8) patients. (B) The correlation analysis of the expression of MEOX1 and circABHD3 in liver samples from hepatic fibrosis patients. (C and D) The expression of MEOX1 in control and TGF-β1-treated THLE-2 and AML12 cells was examined with qRT-PCR and Western blotting (n = 3). (E) Two potential binding sites for MEOX1 in the promoter of ABHD3. (F and G) MEOX1 was overexpressed or knocked down in THLE-2 and AML12 cells, and its expression was examined by qRT-PCR and Western blotting (n = 3). (H and I) The luciferase activity of Site1, Site2, ABHD3 pro-WT or ABHD3 pro-MUT reporters in THLE-2 and AML12 cells transfected with vector or MEOX1 (n = 3). (J) The enrichment of ABHD3 promoter by the MEOX1 antibody was determined with ChIP assays (n = 3). (K) Primers for ABHD3 pre-RNA, mRNA and circABHD3. (L) qRT-PCR analysis of ABHD3 pre-RNA, mRNA and circABHD3 in cells transfected with vector, MEOX1, shNC or shMEOX1 (n = 3). *P < 0.05, **P < 0.01 and ***P < 0.001.

Given the significant involvement of MEOX1 in fibrosis, we examined MEOX1 expression in hepatic fibrosis tissues from patients. MEOX1 was highly expressed, and its expression was positively correlated with circABHD3 expression in hepatic fibrosis tissues (Fig 3A **and** 3B). TGF-β1 treatment significantly upregulated MEOX1 expression at both the mRNA and protein levels in hepatocyte cells (Fig 3C **and** 3D). As mentioned above, circABHD3 promotes EMT and mitochondrial impairment in hepatocytes. However, the mechanisms underlying circABHD3 upregulation remain unclear. It is known that circRNAs are transcribed, spliced, and subsequently cyclized from host genes, and host genes promote circRNA generation [33,34]. Host genes can produce circRNAs through back-splicing of exons. Moreover, the 3′-untranslated regions (3′ UTRs) of cognate mRNAs can be bound by other regulators such as ZC3H14 to promote circRNA biogenesis [35]. Therefore, elucidating the mechanisms of ABHD3 transcription may contribute to understanding circABHD3 generation. Intriguingly, we identified two potential binding sites (Site1 and Site2) for MEOX1 in the promoter region of ABHD3 (Fig 3E). MEOX1 was overexpressed or knocked down in THLE-2 and AML12 cells (Fig 3F **and** 3G). Overexpression of MEOX1 enhanced the luciferase activity of the Site2 reporter but did not affect the luciferase activity of the Site1 reporter (Fig 3H). Besides, overexpression of MEOX1 enhanced the luciferase activity of the ABHD3 pro-WT reporter but did not affect the luciferase activity of the ABHD3 pro-MUT reporter (Fig 3I). Furthermore, the ABHD3 promoter was efficiently enriched by the antibody against MEOX1 (Fig 3J). As shown in Fig 3K, we designed primers to examine the expression of pre-ABHD3, ABHD3 and circABHD3, and they were all upregulated by overexpression of MEOX1 (Fig 3L). Moreover, silencing of MEOX1 significantly reduced the expression of pre-ABHD3, ABHD3 and circABHD3 (Fig 3L). These observations suggested that MEOX1 might promote the expression of ABHD3 mRNA by binding to the ABHD3 promoter, consequently increasing circABHD3 in hepatic fibrosis.

### MEOX1 knockdown inhibited EMT and mitochondrial impairment in TGF-β1-treated THLE-2 and AML12 cells through suppression of circABHD3

**Fig 4:**
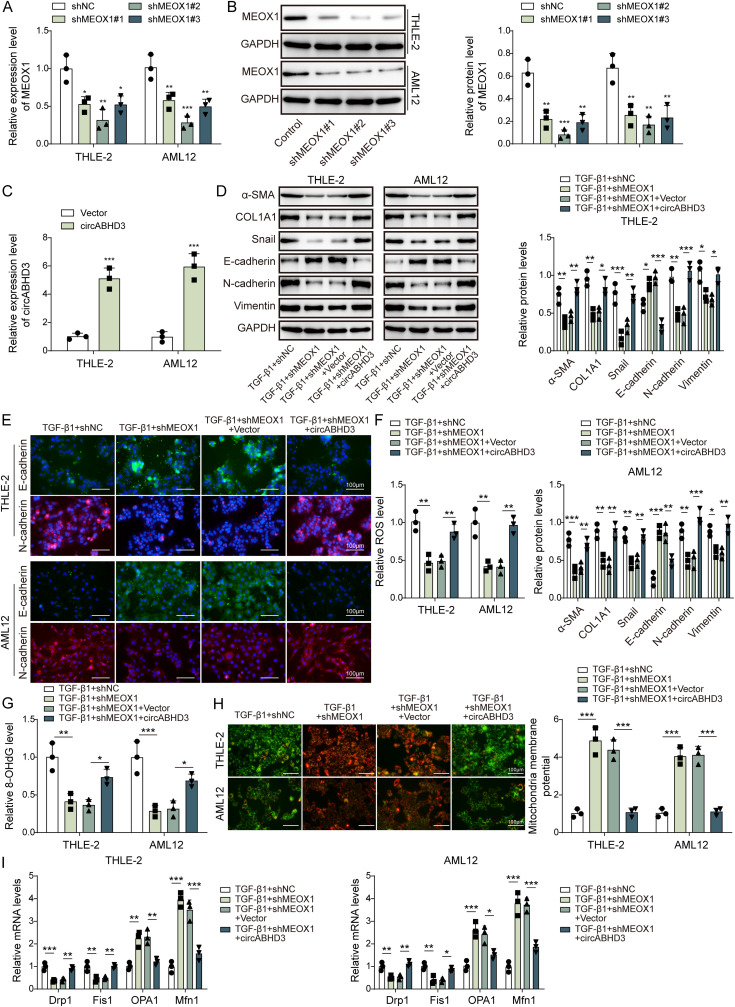
MEOX1 knockdown inhibited EMT and mitochondrial impairment in TGF- **β1-treated THLE-2 and AML12 cells.** MEOX1 was knocked down and circABHD3 was overexpressed in THLE-2 and AML12 cells, and cells were treated with with TGF-β1 at 5 ng/mL for 48 h. (A and B) THLE-2 and AML12 cells were transfected with shNC, shMEOX1#1, shMEOX1#2 or shMEOX1#3, and MEOX1 was detected via qRT-PCR and Western blotting (n = 3). (C) THLE-2 and AML12 cells were transfected with Vector or circABHD3, and circABHD3 was examined by qRT-PCR (n = 3). (D) Protein levels of α-SMA, COL1A1, Snail, E-cadherin, N-cadherin, and Vimentin were detected with Western blotting (n = 3). GAPDH was a loading control for normalization. (E) IF staining of E-cadherin (green) and N-cadherin (red). DAPI was used to stain the nuclei (blue). Scale bar, 100 µm. (F) The ROS level was determined (n = 3). (G) The level of 8-OHdG was examined (n = 3). (H) Mitochondrial membrane potential was examined with JC-1 staining (n = 3). JC-1 monomer, green; JC-1 aggregate, red. Scale bar, 100 µm. (I) The expression of Drp1, Fis1, OPA1 and Mfn1 was analyzed by qRT-PCR (n = 3). *P < 0.05 and **P < 0.01.

To evaluate whether MEOX1 regulates TGF-β1-induced EMT and mitochondrial imbalance through circABHD3, MEOX1 was knocked down and circABHD3 was overexpressed in THLE-2 and AML12 cells, which was confirmed by qRT-PCR and Western blotting (Fig 4A**-**C). TGF-β1-induced upregulation of α-SMA, COL1A1, Snail, N-cadherin and Vimentin and downregulation of E-cadherin were obstructed by MEOX1 knockdown, but simultaneous overexpression of circABHD3 restored their expression (Fig 4D). IF staining showed that TGF-β1-induced E- to N-cadherin transition was inhibited by MEOX1 knockdown, which was reversed by overexpression of circABHD3 (Fig 4E). Furthermore, increased levels of ROS and 8-OHdG and impaired mitochondrial membrane potential in TGF-β1-treated THLE-2 and AML12 cells were reduced by MEOX1 knockdown, and overexpression of circABHD3 abolished MEOX1 knockdown-mediated effects (Fig 4F-H). Besides, knockdown of MEOX1 reduced the expression of Drp1 and Fis1 but enhanced the expression of OPA1 and Mfn1 in TGF-β1-treated THLE-2 and AML12 cells, but their expression was reversed by circABHD3 overexpression (Fig 4I). Thus, these data suggested that knockdown of MEOX1 suppressed TGF-β1-induced EMT and mitochondrial impairment dependent on circABHD3 in THLE-2 and AML12 cells.

### CircABHD3 destabilized YPEL3 mRNA to activate β-catenin signaling in TGF-β1-treated THLE-2 and AML12 cells

**Fig 5:**
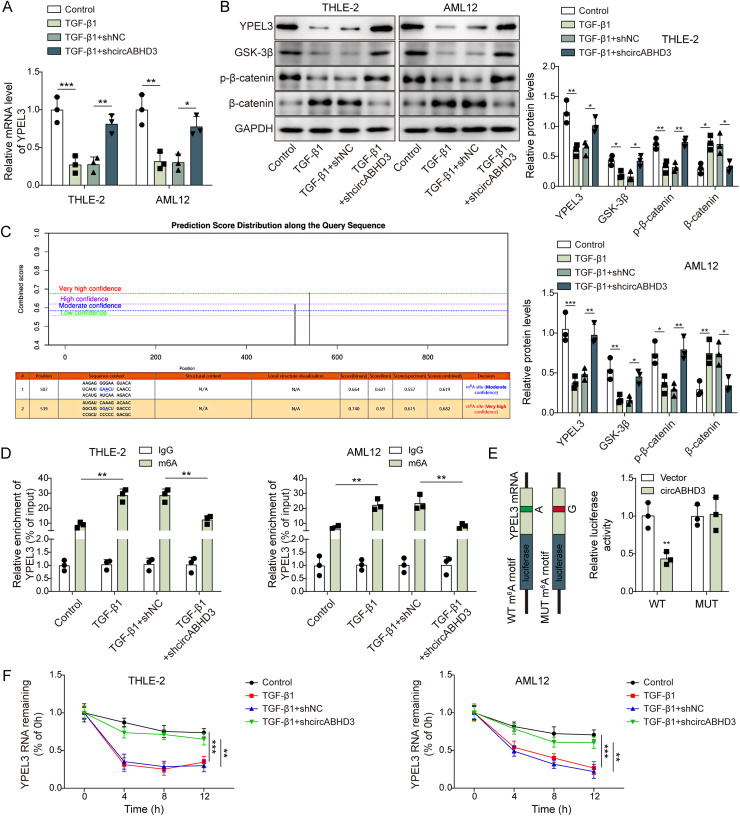
CircABHD3 destabilized YPEL3 mRNA to activate **β-catenin signaling in TGF-β1-treated THLE-2 and AML12 cells.** THLE-2 and AML12 cells were treated with TGF-β1 at 5 ng/mL for 48 h and divided into four groups: Control, TGF-β1, TGF-β1 + shNC and TGF-β1 + shcircABHD3. (A) Relative mRNA levels of YPEL3 were examined by qRT-PCR (n = 3). (B) Protein levels of YPEL3, GSK-3β, total and phosphorylated β-catenin were detected with Western blotting (n = 3). GAPDH was a loading control for normalization. (C) m6A modification sites on YPEL3 mRNA was analyzed by SRAMP database analysis. (D) MeRIP assays for analyzing m6A modification of YPEL3 mRNA (n = 3). (E) The luciferase activity of YPEL3 reporters in THLE-2 and AML12 cells transfected with vector or circABHD3 (n = 3). (F) Remaining YPEL3 mRNA at 0, 4, 8 and 12 h in THLE-2 and AML12 cells after actinomycin D treatment was analyzed by qRT-PCR (n = 3). *P < 0.05, **P < 0.01 and ***P < 0.001.

As YPEL3 can be induced by DNA damage and YPEL3 inhibits EMT of nasopharyngeal carcinoma cells via Wnt/β-catenin signaling [17,36], However, the role of YPEL3 and its regulation of EMT in liver fibrosis was unknown. Thus, to investigate the mechanism by which circABHD3 regulates EMT in liver fibrosis, we decided to examine its expression in TGF-β1-treated THLE-2 and AML12 cells. We observed that YPEL3 was downregulated in TGF-β1-treated THLE-2 and AML12 cells, but the suppressive effect was abrogated by circABHD3 knockdown (Fig 5A **and** 5B), suggesting the involvement of circABHD3 in the regulation of TGF-β1-induced downregulation of YPEL3. As YPEL3 suppresses EMT through the Wnt/β-catenin signaling pathway [17], we examined the levels of GSK-3β, a negative regulator of the Wnt/β-catenin signaling pathway, β-catenin and phosphorylated β-catenin. The levels of GSK-3β and β-catenin phosphorylation were decreased, but the abundance of total β-catenin was increased in response to TGF-β1 treatment, whereas circABHD3 knockdown reversed these effects (Fig 5B), suggesting that circABHD3 knockdown blocked TGF-β1-mediated activation of β-catenin signaling. Additionally, we analyzed the m6A modification of YPEL3 mRNA, as it is widely known to be involved in mRNA degradation. We found two m6A modification sites on YPEL3 mRNA by SRAMP database analysis (with the highest confidence in its A539 site, Fig 5C). The treatment with TGF-β1 led to an increase in m6A modification levels of YPEL3 mRNA in hepatocyte cells (Fig 5D). Overexpression of circABHD3 reduced YPEL3 mRNA luciferase activity, whereas it had no significant effect on YPEL3 mRNA luciferase activity with the A539 site mutation (Fig 5E). The treatment with TGF-β1 significantly reduced the stability of YPEL3 mRNA (Fig 5F). TGF-β1-induced m6A modification of YPEL3 mRNA and subsequent decreased YPEL3 RNA stability were blocked by knockdown of circABHD3 (Fig 5D **and** 5F). Thus, circABHD3 might destabilize YPEL3 mRNA and inhibit its expression, thereby activating β-catenin signaling in hepatic fibrosis.

### CircABHD3 promoted YTHDF2-dependent recognition of m6A-modified YPEL3 mRNA to destabilize YPEL3 mRNA and reduced the expression of YPEL3 and GSK-3β

**Fig 6:**
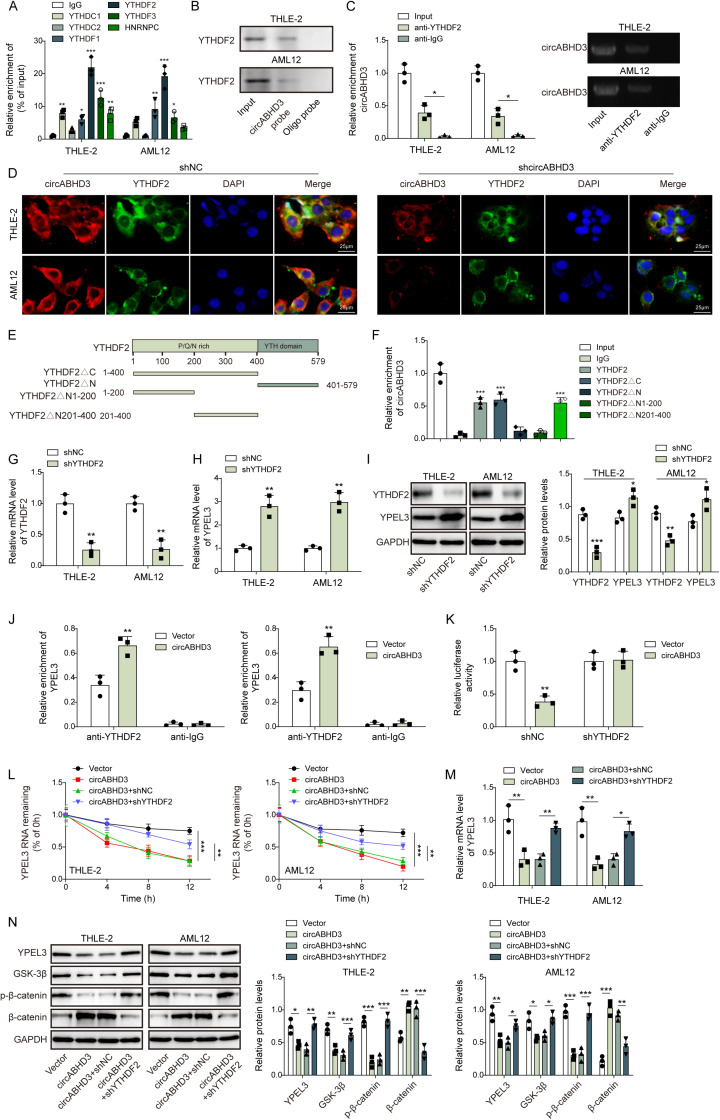
CircABHD3 promoted YTHDF2-dependent recognition of m6A-modified YPEL3 mRNA to destabilize YPEL3 mRNA and reduced the expression of YPEL3 and GSK-3 **β**. (A) The enrichment of YPEL3 mRNA by anti-YTHDC1, anti-YTHDC2, anti-YTHDF1, anti-YTHDF2, anti-YTHDF3 and anti-HNRNPC was determined by RIP-qPCR analysis (n = 3). Normal IgG was used as the negative control. (B) YTHDF2 was pulled down by the circABHD3 probe and subjected for electrophoresis. (C) CircABHD3 was enriched by an YTHDF2 antibody and subjected to qRT-PCR and electrophoresis (n = 3). (D) Colocalization analysis of circABHD3 and YTHDF2 through combined FISH and IF staining in shNC and shcircABHD3-transfected THLE-2 and AML12 cells. DAPI was used to stain the nuclei (blue). Scale bar, 25 µm. (E) Various truncated constructs of YTHDF2 including YTHDF2ΔC, YTHDF2ΔN, YTHDF2ΔN1-200 and YTHDF2ΔN201-400. (F) CircABHD3 was enriched and subjected to qRT-PCR (n = 3). (G and H) YTHDF2 was knocked down in THLE-2 and AML12 cells, and relative mRNA levels of YTHDF2 and YPEL3 were analyzed by qRT-PCR (n = 3). (I) Western blot analysis of YTHDF2 and YPEL3 (n = 3). GAPDH was a loading control for normalization. (J) THLE-2 and AML12 cells were transfected with vector or circABHD3, and the enrichment of YPEL3 by the YTHDF2 antibody was analyzed by RIP-qPCR (n = 3). THLE-2 and AML12 cells were divided into four groups: Vector, circABHD3, circABHD3 + shNC and circABHD3 + shYTHDF2. (K) The luciferase activity of YPEL3 reporters in THLE-2 and AML12 cells transfected with circABHD3 or shYTHDF2 (n = 3). (L) Remaining YPEL3 mRNA at 0, 4, 8 and 12 h after actinomycin D treatment was analyzed by qRT-PCR (n = 3). (M) Relative mRNA levels of YPEL3 were examined by qRT-PCR (n = 3). (N) Protein levels of YPEL3, GSK-3β, phosphorylated and total β-catenin were detected with Western blotting (n = 3). GAPDH was a loading control for normalization. *P < 0.05, **P < 0.01 and ***P < 0.001.

The level of m6A is primarily modulated by m6A writers and erasers, while m6A readers can recognize m6A and regulate mRNA translation and stability [37]. Therefore, we assessed the enrichment of YPEL3 mRNA by employing antibodies that specifically target various m6A reader proteins, such as YTHDC1, YTHDC2, YTHDF1, YTHDF2, YTHDF3, and HNRNPC. The IP efficiencies among anti-YTHDC1, anti-YTHDC2, anti-YTHDF1, anti-YTHDF2, anti-YTHDF3 and anti-HNRNPC were almost similar (S 2A Fig). Our results indicated that the YTHDF2 antibody exhibited the greatest enrichment of YPEL3 mRNA (Fig 6A). CircABHD3 rather than ABHD3 and GAPDH mRNA could be efficiently pulled down by the circABHD3 probe (S2B Fig). The results of circABHD3 pull-down assay demonstrated the presence of YTHDF2 in the products pulled down by the circABHD3 probe (Fig 6B). The YTHDF2 antibody RIP experiment also confirmed the binding relationship between YTHDF2 and circABHD3 (Fig 6C), and no significant co-immunoprecipitation of ABHD3 mRNA was observed (S3 Fig). Furthermore, FISH and IF co-staining in THLE-2 and AML12 cells proved the co-localization relationship of circABHD3 and YTHDF2, and the signal of circABHD3 was dramatically reduced after circABHD3 knockdown (Fig 6D). These data support the hypothesis that circABHD3 may participate in YPEL3 mRNA stability regulation through its interaction with YTHDF2. YTHDF2 consists of a C-terminal YTH structural domain responsible for binding to m6A and an N-terminal structural domain enriched with P/Q/N (Fig 6E). The main function of the C-terminal YTH structural domain is to recognize and bind to m6A-modified RNA molecules, and to participate in the regulation of the metabolism of RNAs, including mRNA translation and stability. We constructed different truncated vectors of YTHDF2 to determine the specific binding for sites of circABHD3 and YTHDF2 (Fig 6E). The results showed that circABHD3 was significantly enriched by YTHDF2, YTHDF2ΔC and YTHDF2ΔN201-400 rather than YTHDF2ΔN and YTHDF2ΔN1-200 (Fig 6F), suggesting that circABHD3 primarily bound to 1-200 in the N-terminal region of YTHDF2. Subsequently, to investigate the regulation of YPEL3 mRNA stability by YTHDF2, YTHDF2 was knocked down in THLE-2 and AML12 cells, and we observed a significant increase in YPEL3 expression (Fig 6G–I). Furthermore, YPEL3 mRNA could be enriched by the YTHDF2 antibody, and circABHD3 overexpression enhanced the enrichment (Fig 6J). Knockdown of YTHDF2 impaired the inhibitory effect of circABHD3 overexpression on YPEL3 mRNA luciferase activity (Fig 6K). Overexpression of circABHD3 accelerated the decay of YPEL3 mRNA to reduce YPEL3 expression, but it was reversed by YTHDF2 knockdown (Fig 6L **and** 6M). In addition, overexpression of circABHD3 reduced the protein levels of YPEL3, GSK-3β and phosphorylated β-catenin and promoted the expression of total β-catenin in THLE-2 and AML12 cells, but knockdown of YTHDF2 reversed their expression patterns (Fig 6N). Therefore, we concluded that circABHD3 promoted YTHDF2 binding to YPEL3 to facilitate YTHDF2-mediated YPEL3 mRNA decay, subsequently reducing the expression of YPEL3 and GSK-3β and leading to the activation of β-catenin signaling.

### Knockdown of YPEL3 or activation of β-catenin reversed circABHD3 silencing-mediated prevention of EMT and mitochondrial impairment in TGF-β1-induced hepatocytes

**Fig 7:**
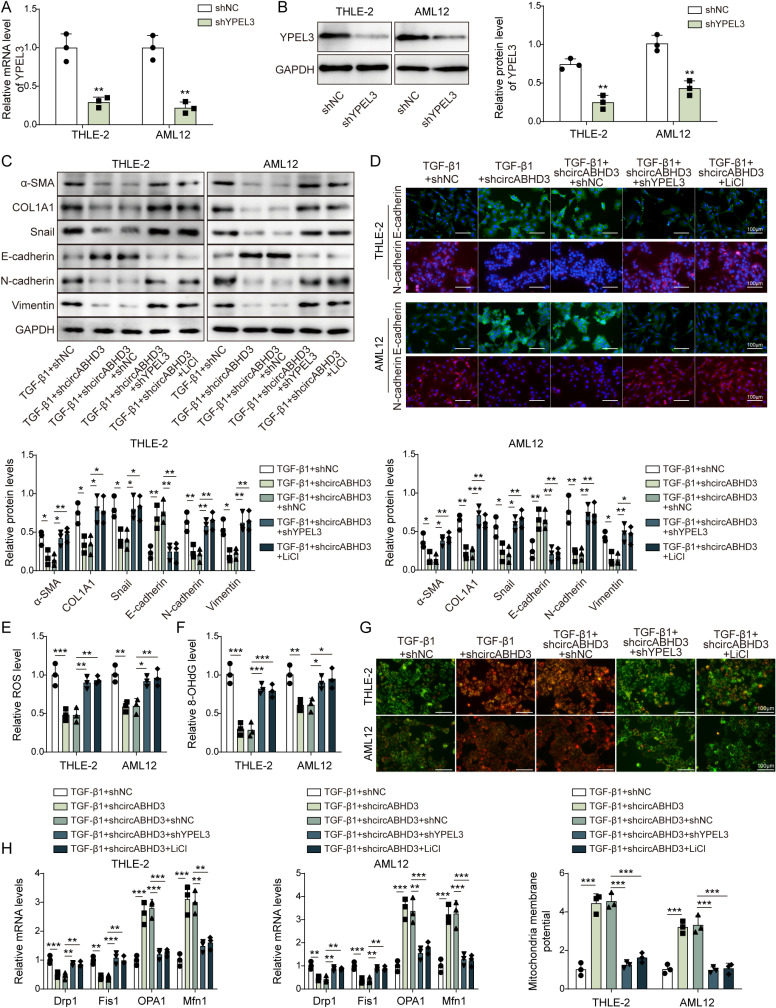
Knockdown of YPEL3 or activation of **β-catenin reversed circABHD3 silencing-mediated prevention of EMT and mitochondrial impairment in TGF-β1-induced hepatocytes.** (A and B) Relative mRNA and protein levels of YPEL3 in shNC or shYPEL3-transfected THLE-2 and AML12 cells were examined with qRT-PCR and Western blotting (n = 3). THLE-2 and AML12 cells were divided into five groups: TGF-β1 + shNC, TGF-β1 + shcircABHD3, TGF-β1 + shcircABHD3 + shNC, TGF-β1 + shcircABHD3 + shYPEL3 and TGF-β1 + shcircABHD3 + LiCl. (C) Western blot analysis of α-SMA, COL1A1, Snail, E-cadherin, N-cadherin, and Vimentin (n = 3). GAPDH was a loading control for normalization. (D) IF staining of E-cadherin (green) and N-cadherin (red) for evaluating cadherin switching (n = 3). DAPI was used to stain the nuclei (blue). Scale bar, 100 µm. (E) The ROS level was determined (n = 3). (F) The level of 8-OHdG was examined (n = 3). (G) Mitochondrial membrane potential was examined with JC-1 staining (n = 3). JC-1 monomer, green; JC-1 aggregate, red. Scale bar, 100 µm. (H) The expression of Drp1, Fis1, OPA1 and Mfn1 was analyzed by qRT-PCR (n = 3). *P < 0.05, **P < 0.01 and ***P < 0.001.

YPEL3 was knocked down in THLE-2 and AML12 cells, and its knockdown was confirmed by qRT-PCR and Western blotting (Fig 7A **and** 7B). THLE-2 and AML12 cells were transfected with shcircABHD3 and shYPEL3 and treated with lithium chloride (LiCl) for induce activation of β-catenin signaling. Cells were divided into TGF-β1 + shNC, TGF-β1 + shcircABHD3, TGF-β1 + shcircABHD3 + shNC, TGF-β1 + shcircABHD3 + shYPEL3 and TGF-β1 + shcircABHD3 + LiCl groups. The results demonstrated that silencing of circABHD3 significantly reduced the expression of α-SMA, COL1A1, Snail, N-cadherin and Vimentin and upregulated E-cadherin, but these effects were reversed by YPEL3 knockdown or LiCl treatment (Fig 7C **and** 7D). Besides, circABHD3 knockdown-mediated suppressive effects on the levels of ROS and 8-OHdG and mitochondrial membrane potential depolarization in TGF-β1-treated THLE-2 and AML12 cells were abolished by YPEL3 knockdown or LiCl treatment (Fig 7E–G). Drp1 and Fis1 were downregulated and OPA1 and Mfn1 were upregulated by circABHD3 knockdown, which was reversed by YPEL3 knockdown or LiCl treatment (Fig 7H). Thus, the relief of TGF-β1-induced EMT and mitochondrial imbalance resulting from circABHD3 silencing was dependent on YPEL3 and subsequent inhibition of β-catenin signaling.

### Depletion of circABHD3 ameliorated hepatic fibrosis in mice

**Fig 8:**
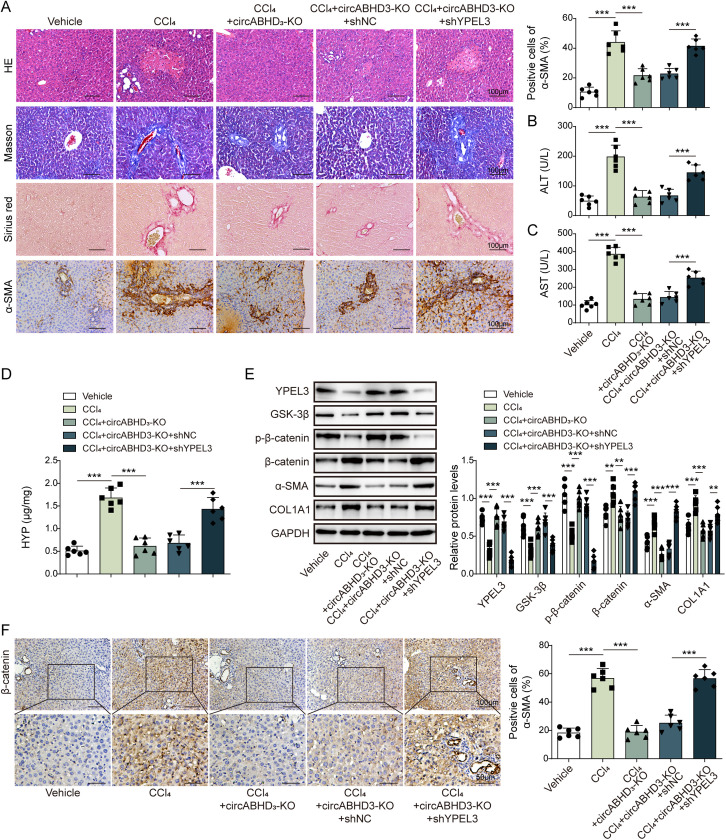
Depletion of circABHD3 ameliorated hepatic fibrosis in mice. Wildtype and circABHD3 knockout (KO) mice were intraperitoneally injected with CCl_4_ for hepatic fibrosis induction and divided into five groups: Vehicle, CCl_4_, CCl_4_ + circABHD3-KO, CCl_4_ + circABHD3-KO + shNC and CCl_4_ + circABHD3-KO + shYPEL3. YPEL3-silencing lentiviral particles were intravenously injected. (A) Liver injury and fibrosis were evaluated by H&E, Masson’s trichrome and Sirius Red staining and α-SMA was detected by IHC staining. Scale bar, 100 µm. (B and C) Serum ALT and AST were determined by ELISA (n = 6). (D) HYP concentration in the livers was determined (n = 6). (E) Protein levels of YPEL3, GSK-3β, α-SMA, COL1A1, total and phosphorylated β-catenin in the livers were examined with Western blotting (n = 6). GAPDH was a loading control for normalization. (F) IHC staining of β-catenin in the livers. Scale bar, 100 µm or 50 µm. **P < 0.01 and ***P < 0.001.

Mice were divided into five groups: Vehicle, CCl_4_, CCl_4_ + circABHD3-KO, CCl_4_ + circABHD3-KO + shNC and CCl_4_ + circABHD3-KO + shYPEL3. CCl_4_ was intraperitoneally injected into mice for liver fibrosis induction. As expected, CCl_4_-treated wildtype mice showed obvious liver injury and increased collagen deposition and fibrotic areas, as exhibited by H&E, Masson’s trichrome and Sirius Red staining, accompanied by an elevation in α-SMA protein levels, demonstrating the effectiveness of the liver fibrosis induction in mice (Fig 8A). However, no significant CCl_4_-induced damage was observed in circABHD3 knockout mice (Fig 8A). Importantly, circABHD3 depletion-mediated protection was abolished by YPEL3 knockdown (Fig 8A). Meanwhile, the pathological elevation of serum ALT, AST, and hydroxyproline (HYP) induced by CCl_4_ treatment were significantly blocked by circABHD3 knockout, but these protective effects were reversed by YPEL3 knockdown (Fig 8B–D). CCl_4_-treated mice showed decreased expression of YPEL3, GSK-3β and phosphorylated β-catenin and elevated expression of total β-catenin, α-SMA and COL1A1 in the livers, whereas depletion of circABHD3 reversed their expression, and knockdown of YPEL3 abolished these effects (Fig 8E **and** 8F). Thus, depletion of circABHD3 suppressed CCl_4_-induced hepatic fibrosis dependent on YPEL3 upregulation and subsequent suppression of β-catenin signaling in mice.

### Depletion of circABHD3 inhibited CCl _4_ -induced EMT and mitochondrial impairment dependent on YPEL3 in mice

**Fig 9:**
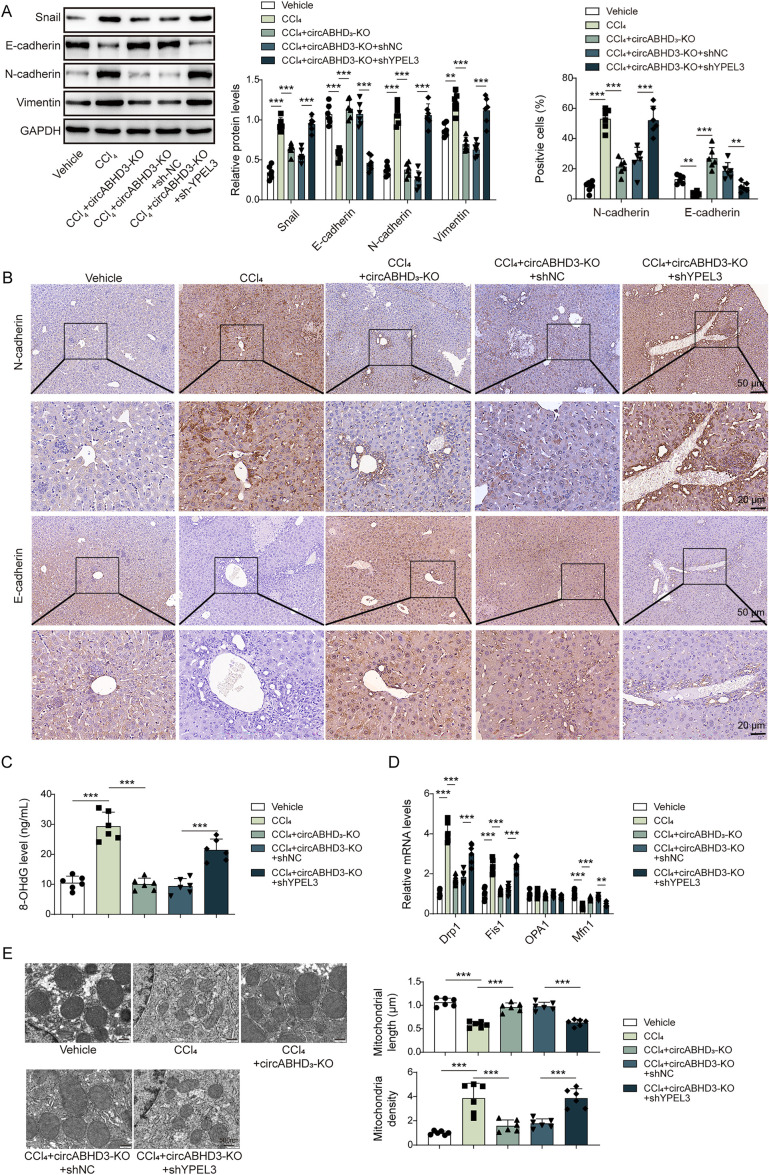
Depletion of circABHD3 inhibited CCl_4_-induced EMT and mitochondrial impairment in mice. (A) Western blot analysis of Snail, E-cadherin, N-cadherin, and Vimentin in the livers (n = 6). (B) IHC staining of N- and E-cadherin in the livers. Scale bar, 50 µm or 20 µm. (C) Serum 8-OHdG was determined by ELISA. (D) Relative mRNA levels of Drp1, Fis1, OPA1 and Mfn1 were analyzed by qRT-PCR (n = 6). (E) Mitochondrial morphology and fission were determined with TEM. Scale bar, 500 nm. *P < 0.05, **P < 0.01 and ***P < 0.001.

We further examined the expression of EMT-related markers and found that the expression of Snail, Vimentin and N-cadherin were enhanced and E-cadherin was downregulated in the livers from CCl_4_-treated mice, but these changes were reversed by circABHD3 depletion, and circABHD3 depletion-mediated protective effects on CCl_4_-induced EMT was abrogated by YPEL3 knockdown (Fig 9A **and** 9B). Depletion of circABHD3 reduced the levels of 8-OHdG, Drp1 and Fis1 upregulated OPA1 and Mfn1 in CCl_4_-treated mice, which was abolished by YPEL3 knockdown (Fig 9C **and** 9D). In addition, CCl_4_-treated mice showed shortened mitochondrial length and increased mitochondrial density, and these changes were inhibited by depletion of circABHD3 (Fig 9E). However, depletion of circABHD3-mediated effects on mitochondrial morphology and fission were reversed by YPEL3 knockdown (Fig 9E). We also established the bile duct ligation (BDL) model of hepatic fibrosis and found that BDL mice showed obvious liver injury and increased collagen deposition, fibrotic areas and α-SMA, which were reversed by knockdown of circABHD3 (S4A Fig). Increased levels of ALT, AST and HYP in BDL mice were reduced by circABHD3 depletion (S4B-D Fig). Increased ratio of N-cadherin-positive cells and decreased ratio of E-cadherin-positive cells in BDL mice were reduced by depletion of circABHD3 (S4E Fig). Collectively, these findings implied that depletion of circABHD3 inhibited CCl4-induced EMT and mitochondrial impairment through YPEL3 *in vivo*.

## Discussion

Although it has few symptoms, hepatic fibrosis is still a serious health issue worldwide as it results in liver cirrhosis and carcinoma [38]. Liver cirrhosis is the end stage of fibrosis, and around one million deaths a year are caused by liver cirrhosis and its complications globally [39]. Fortunately, growing evidence suggests that hepatic fibrosis is reversible in both patients and experimental fibrotic models [40,41]. It is of great importance to further understand the pathogenesis of hepatic fibrosis and develop novel medications for hepatic fibrosis management. Here, we found that circABHD3 exacerbated hepatic fibrosis via promoting EMT and mitochondrial impairment in liver fibrosis. Mechanically, circABHD3 destabilized YPEL3 mRNA through promotion of YTHDF2-mediated degradation of YPEL3 mRNA, thus activating β-catenin signaling. In addition, MEOX1 could bound to the promoter of ABHD3 to enhance its transcription and subsequent circABHD3 generation.

The onset and progression of hepatic fibrosis are quite complicated processes, in which EMT has been shown to be a contributor in many studies [12]. Zeisberg et al. demonstrated that hepatocyte-derived fibroblasts via undergoing EMT promoted the progression of hepatic fibrosis, supporting the concept that EMT in hepatocytes contributes to hepatic fibrosis [42]. However, several studies suggest that the roles of EMT in hepatic fibrosis remain under debate [43]. A study challenged the notion that hepatocytes underwent EMT to generate ECM proteins in hepatic fibrosis [44]. Chu et al. found that cholangiocytes and hepatocytes did not undergo EMT in liver fibrosis mouse models [45]. Further extensive investigations need to be conducted to reconcile these conflicting data. Our findings support the concept that EMT contributes to hepatic fibrosis, and blockade of EMT may be a therapeutic strategy for fibrosis intervention.

CircRNAs are emerging as important regulators in EMT and hepatic fibrosis. circUHRF1 was reported to enhance EMT in oral squamous cell carcinoma [46]. Xu et al. found that hsa_circ_0003288 promoted EMT in liver cancer [47]. Several circRNAs are implicated in the progression of hepatic fibrosis [48]. Wang et al. reported that circMTO1 suppressed hepatic fibrosis by modulating miR‐17‐5p and Smad7 [49]. Zhu and colleagues proved that circUbe2k enhanced liver fibrosis through the miR-149-5p/TGF-β2 axis [50]. However, circRNA-mediated linkage between EMT and hepatic fibrosis remains largely unknown. In our study, we found that circABHD3 was highly expressed in hepatic fibrosis tissues and models, and circABHD3 exacerbated CCl_4_-induced liver injury and fibrosis through promotion of EMT *in vivo*, suggesting a novel mechanism underlying circRNA-mediated regulation of hepatic fibrosis via EMT. Besides, the upstream regulatory mechanisms underlying circRNA generation are key to understand the roles of circRNAs in various pathological conditions. CircRNAs are derived from host genes, and host genes facilitate corresponding circRNA generation by back-splicing involving other regulators such as ZC3H14-mediated promotion of circRNA biogenesis by facilitating back-splicing, thus producing a positive feedback loop for the regulation of circRNAs and host genes [33, 34]. We identified a novel regulatory mechanism of circABHD3 generation that MEOX1 can bind to ABHD3 promoter to facilitate ABHD3 transcription and circABHD3 generation, thus exacerbates EMT and mitochondrial impairment through circABHD3. Emerging evidence has suggested that many circRNAs are differentially expressed in liver fibrotic tissues, and circRNAs are highly abundant in blood [51,52]. Thus, circRNAs may be developed as reliable biomarkers of diseases and treatment efficacy due to their highly stable nature and remarkable tissue specificity. Our findings provide evidence for circABHD3 as a potential diagnostic and prognostic markers and therapeutic target for liver fibrosis.

YTHDF2, a m6A reader, enhances the degradation of mRNAs via binding to m6A-modified mRNAs [53,54]. For example, YTHDF2 targets and destabilizes m6A-modified mRNAs such as neural-specific genes [55]. Besides, YTHDF2 also regulates mRNA decay through other mechanisms such as 5′-to-3′ decay and internal cleavage, and YTHDF2 triggers rapid degradation of m6A-containing mRNAs by recognizing m6A and recruiting RNA-degrading enzymes or adaptor proteins [56,57]. A preprint reported that METTL3 promoted the progression of hepatic fibrosis through YTHDF2-mediated silencing of GPR161 in a m6A-dependent manner [58]. Reduced EMT was observed in YTHDF2-deleted Hela cells [59], and overexpression of YTHDF2 enhanced EMT in lung squamous cell carcinoma cells [60]. Consistently, for the first time, we confirmed that YTHDF2 recognized m6A-YPEL3 mRNA to enhance its degradation. Therefore, circABHD3 destabilized YPEL3 mRNA through YTHDF2 in a m6A-dependent manner, thus promoting EMT and hepatic fibrosis.

The β-catenin signaling contributes to the initiation of EMT via its nuclear translocation and regulating the expression of downstream target genes in various fibrotic diseases [61,62]. β-catenin can be phosphorylated by GSK-3β for ubiquitin-mediated degradation that is vital for suppressing the activation of β-catenin signaling [63]. In addition, YPEL3, a member of the putative zinc finger motif, is emerging as a negative regulator of β-catenin signaling to suppress EMT in cancer [17]. YPEL3 has been reported to inhibit tumor proliferation, metastasis and growth [64], but its roles in hepatic fibrosis are unknown. We found that circABHD3 facilitated the decay of YPEL3 mRNA in a m6A/YTHDF2 dependent manner, leading to GSK-3β downregulation and inactivation of β-catenin signaling in liver fibrosis.

Taken together, we firstly demonstrated that MEOX1-regulated circABHD3 exacerbated hepatic fibrosis via promoting EMT and mitochondrial through suppression of YPEL3-mediated inactivation of β-catenin signaling in a YTHDF2-dependent manner. Our results not only deepen understanding of the pathogenesis of hepatic fibrosis but also suggests potential therapeutic targets. However, to do this, more clinical samples should be included in further investigations.

## Supporting information

S1 Fig The level of ABHD3 mRNA was not affected by TGF-β1 or CCl4 treatment. THLE-2 and AML12 cells were treated with TGF-β1 at 5 ng/mL for 48 h and divided into two groups: Control and TGF-β1. (A) qRT-PCR analysis of ABHD3 mRNA in TGF-β1 or vehicle-treated cells (n = 3). Mice were treated with CCl_4_ or vehicle, and qRT-PCR analysis of ABHD3 mRNA in CCl_4_ or vehicle-treated mice (n = 6).
(TIF)

S2 Fig The IP efficiencies of RIP assays and pull-down specificity of RNA pull-down assays were examined. (A) The immunoprecipitated YTHDC1, YTHDC2, YTHDF1, YTHDF2, YTHDF3 and HNRNPC were detected by Western blotting. (B) The abundance of circABHD3, ABHD3 mRNA and GAPDH mRNA pulled down by the circABHD3 probe was examined by qRT-PCR (n = 3). ***P < 0.001.
(TIF)

S3 Fig ABHD3 mRNA was not enriched by an YTHDF2 antibody. The enrichment of ABHD3 mRNA by an YTHDF2 antibody was determined with qRT-PCR and electrophoresis (n = 3).
(TIF)

S4 Fig Depletion of circABHD3 alleviated liver fibrosis in BDL mice. Wildtype and circABHD3 KO mice were induced for hepatic fibrosis with BDL and divided into four groups: Sham-WT, Sham-circABHD3-KO, BDL-WT and BDL-circABHD3-KO. Scale bar, 100 µm. (A) Liver injury and fibrosis were evaluated by H&E, Masson’s trichrome and Sirius Red staining and α-SMA was detected by IHC staining. (B and C) Serum ALT and AST were determined by ELISA (n = 6). (D) HYP concentration in the livers was determined (n = 6). (E) IHC staining of E-cadherin and N-cadherin in the livers. Scale bar, 100 µm or 20 µm. *P < 0.05, **P < 0.01 and ***P < 0.001.
(TIF)

S1 Table Numerical values supporting each graph.
(XLSX)
