# Supplementary figures and images for: MEOX1-mediated transcriptional regulation of circABHD3 exacerbates hepatic fibrosis through promoting m6A/YTHDF2-dependent YPEL3 mRNA decay to activate β-catenin signaling
Source: PLoS Genet. 2025 Mar 18;21(3):e1011622. doi: 10.1371/journal.pgen.1011622 (PMC11918346; doi:10.1371/journal.pgen.1011622)

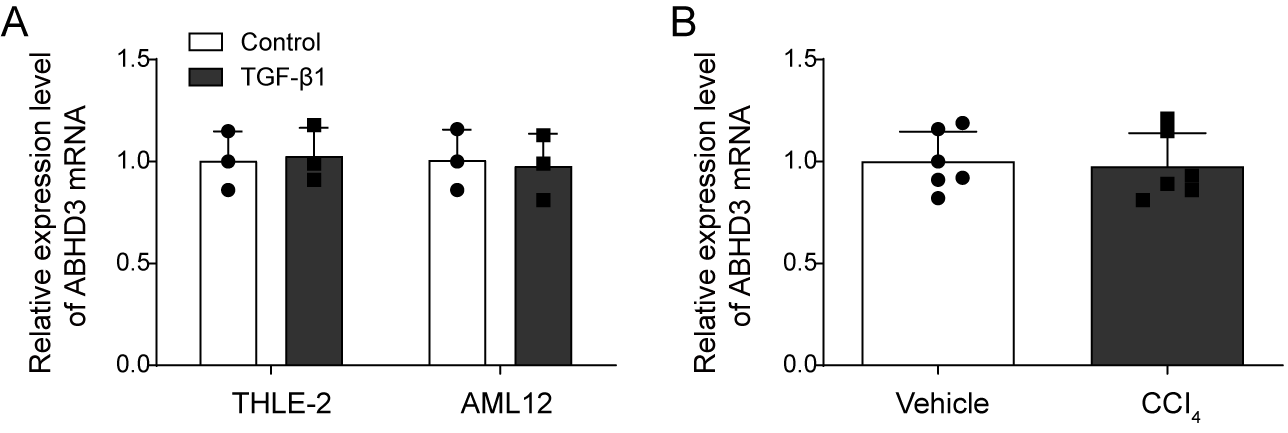

Supplement: S1 Fig — (A) qRT-PCR analysis of ABHD3 mRNA in TGF-β1 or vehicle-treated cells (n = 3). Mice were treated with CCl4 or vehicle, and qRT-PCR analysis of ABHD3 mRNA in CCl4 or vehicle-treated mice (n = 6). (TIF) [file pgen.1011622.s001.tif]

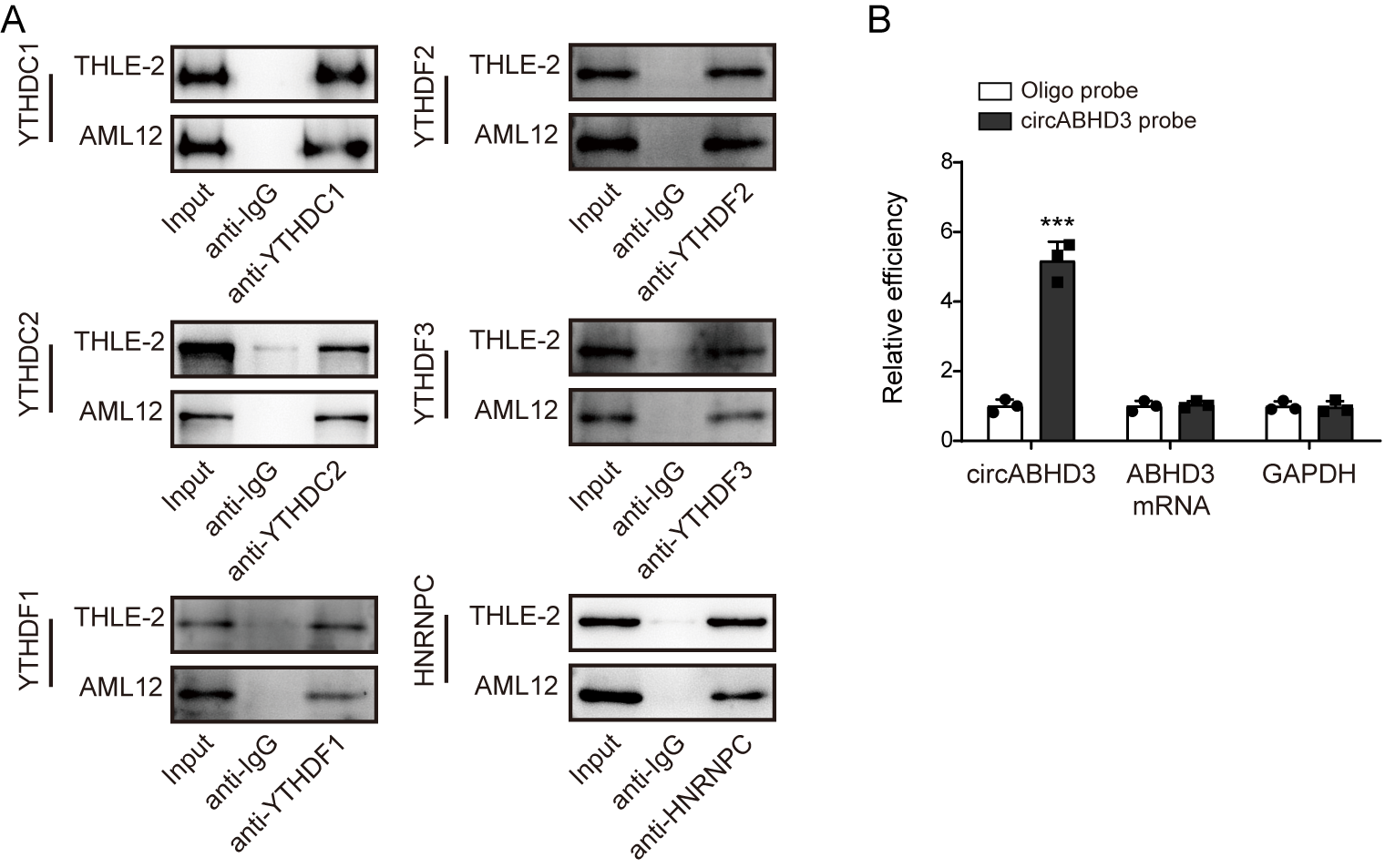

Supplement: S2 Fig — (B) The abundance of circABHD3, ABHD3 mRNA and GAPDH mRNA pulled down by the circABHD3 probe was examined by qRT-PCR (n = 3). ***P < 0.001. (TIF) [file pgen.1011622.s002.tif]

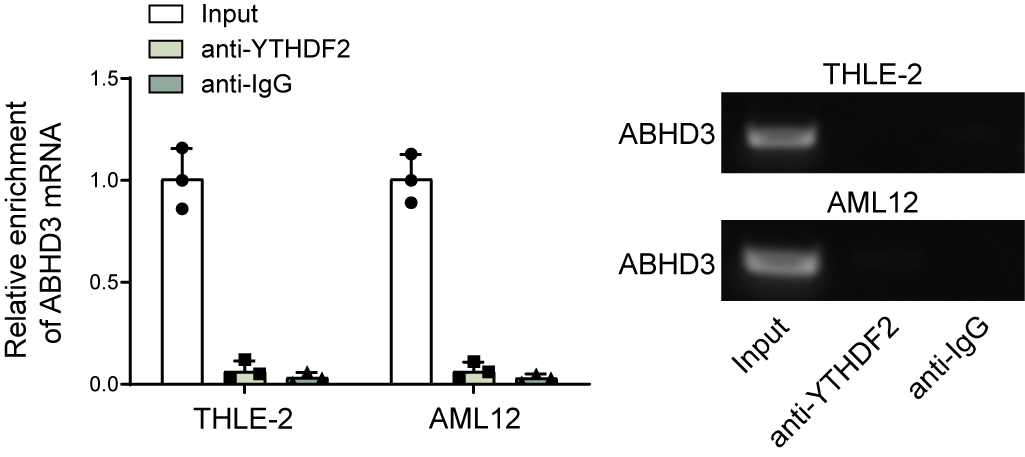

Supplement: S3 Fig — The enrichment of ABHD3 mRNA by an YTHDF2 antibody was determined with qRT-PCR and electrophoresis (n = 3). (TIF) [file pgen.1011622.s003.tif]

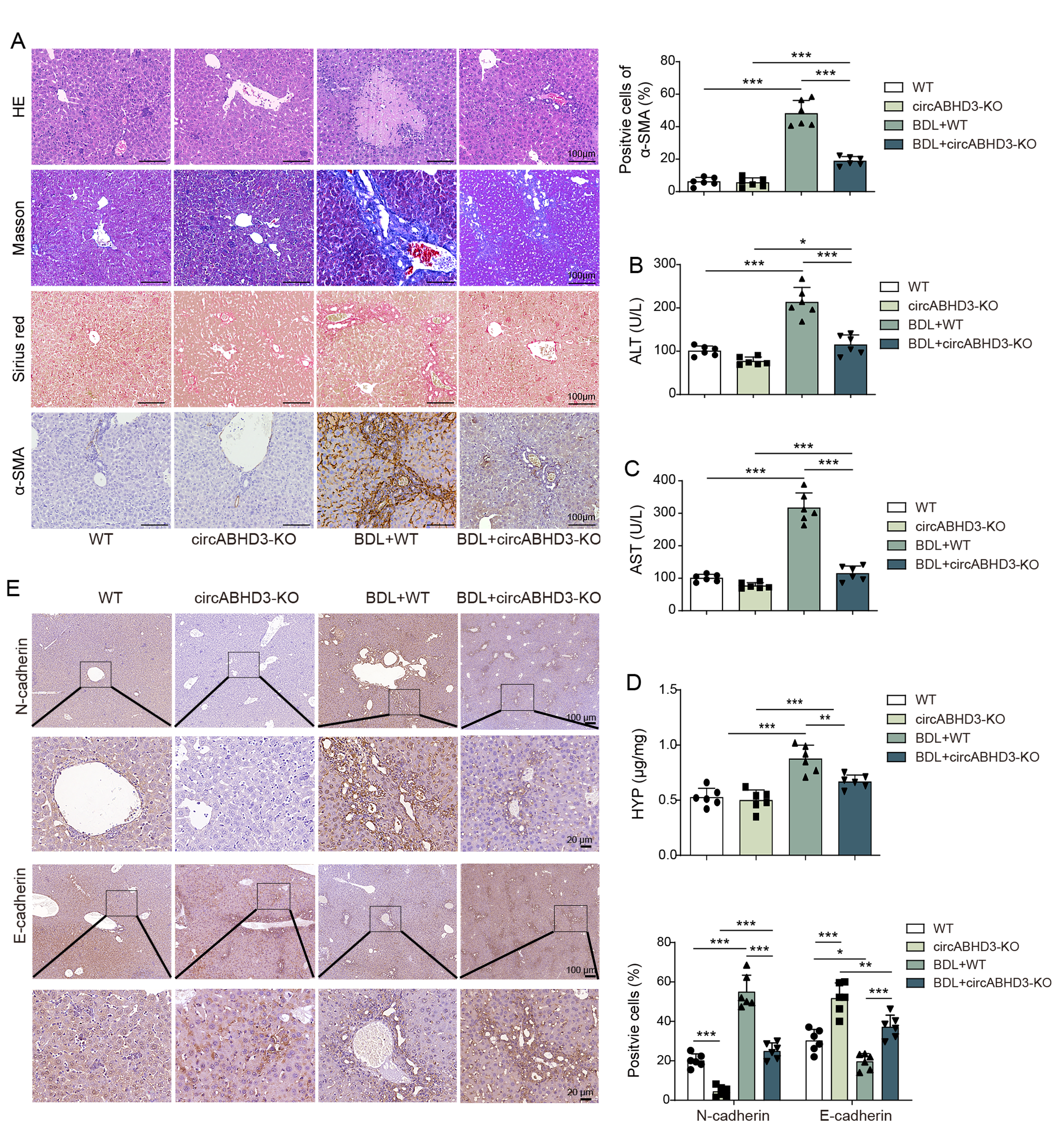

Supplement: S4 Fig — Scale bar, 100 µm. (A) Liver injury and fibrosis were evaluated by H&E, Masson’s trichrome and Sirius Red staining and α-SMA was detected by IHC staining. (B and C) Serum ALT and AST were determined by ELISA (n = 6). (D) HYP concentration in the livers was determined (n = 6). (E) IHC staining of E-cadherin and N-cadherin in the livers. Scale bar, 100 µm or 20 µm. *P < 0.05, **P < 0.01 and ***P < 0.001. (TIF) [file pgen.1011622.s004.tif]
